# Evaluating the implementation of a community engaged telehealth based intervention to improve health equity for the unhoused

**DOI:** 10.3389/fpubh.2025.1487842

**Published:** 2025-05-09

**Authors:** Emily Johnson, Sarah Tucker Marrison, Mattie Banks, Cristin Swords Adams

**Affiliations:** ^1^College of Nursing, Medical University of South Carolina, Charleston, SC, United States; ^2^Department of Family Medicine, Medical University of South Carolina, Charleston, SC, United States

**Keywords:** unhoused, homeless, dissemination implementation, telehealth, healthcare access

## Abstract

Unhoused individuals experience numerous barriers to healthcare access and higher morbidity and mortality rates than housed individuals. In collaboration with community-based organizations (CBOs) and healthcare profession learners we developed a program involving in-person and telehealth visits at a CBO clinic and via street medicine outreach to address healthcare needs of the unhoused in a small Southeastern city. In its fifth year of operation, from January through April 2024, we evaluated the program using key stakeholder interviews (patients, CBO staff learners) guided by the Reach Effectiveness Adoption Implementation Maintenance (RE-AIM) framework. A template analysis approach was utilized to evaluate barriers and facilitators to implementation outcomes. Results demonstrated consistent themes across stakeholders. Factors central to reach included advertisement by word-of-mouth, location convenience, and perceived service benefits. For learners, barriers to reach included clinic hours conflicting with academic schedules and apprehension of providing medical care in this setting. Regarding effectiveness, facilitating themes included opportunities for autonomy and enhanced understanding of treatment of underserved populations (learners) and improvement in health (patients, CBO staff). There were no consistently identified unintended negative consequences of the program. For adoption, all stakeholders described strong perceptions of trust in providers and the importance of team communication and coordination of care, in addition to the need to add disciplines included in the multidisciplinary care team. Facilitating implementation themes included comprehensive access for existing patients, while barriers identified were adequacy of access to social resources (housing, food, transportation) and continued increase in numbers of unhoused individuals outpacing available services. Themes facilitating maintenance included continued outreach efforts and integration into existing healthcare and community-based systems. The addition of additional services and specialties was consistently identified as essential to health status of the patients and an opportunity for growth of the program. This implementation evaluation involving key stakeholders of a community engaged telehealth-based intervention for the unhoused provides thematic considerations to guide program implementation and sustainability to improve health equity for vulnerable populations.

## 1 Introduction

The Institute of Global Homelessness defines homelessness as people without accommodation (sleeping in open or roofed spaced not meant for human habitation), those living in temporary or crisis accommodation (shelters, camps), and those living in severely inadequate and insecure accommodation (temporary sharing with others, overcrowding) (1). Approximately 1.6 billion people worldwide lack adequate housing (2). Unhoused individuals are at significantly higher risk of numerous, complex health problems leading to higher morbidity and mortality than housed individuals. Due to the living conditions of the unhoused, whether living on the street or in a shelter, this population is prone to encounter multiple health risks and negative health outcomes including but not limited to exposure to communicable diseases, poor nutrition, as well as psychological stressors (3). Increased morbidity and mortality are multifactorial but includes higher incidence of chronic medical conditions including cancer and heart disease as well as substance use disorder (4). The unhoused also have high-cost healthcare utilization patterns including lower ambulatory care use and higher emergency department use and hospitalization (5). There are various barriers that contribute to the complexities of providing healthcare to unhoused populations. Common healthcare barriers affecting health care access for the unhoused include unaffordable cost of care, being uninsured, and little to no transportation to obtain health services (3). In order to address the barriers to healthcare access for those who experience homelessness, it is imperative to present accessible health care services that target the needs of this population in order to reduce morbidity and mortality rates (6). Thus, innovative methods of care delivery are key to reaching this population. Utilizing telehealth has been proven as an important factor to help connect vulnerable populations to health care and eliminate barriers for those who are unable to obtain appropriate health care (7, 8). Programs which deliver telehealth to the unhoused are most successful when there is collaboration between agencies for arrangement of health services to meet patient needs (7, 8). Telehealth as a mode of delivery has proved to be effective in providing healthcare access for Veterans experiencing homelessness, with telehealth tablets being used to overcome health and travel related barriers (7). However, there are limited published implementation evaluations related to telehealth delivery in the unhoused population. The Reach Effectiveness Adoption Implementation Maintenance (RE-AIM) framework is used to evaluate program implementation by assessing program internal and external validity to enhance the sustainability and generalizability of interventions. It is a useful tool for these evaluations as it can guide modifications or replications of effective interventions and is practical for local settings while focusing on the multi- dimensional impact related to population health outcomes. Its components include Reach (program contact with target population), Effectiveness (impact of the program on outcomes), Adoption (willingness of relevant individuals and groups to initiate the program), Implementation (consistency of programmatic elements with the intended program including resource utilization and adaptations), Maintenance (integration of the program into routine practice) (9). The RE-AIM framework has previously been utilized to evaluate telehealth within primary care during the COVID-19 pandemic as well as implementation of telehealth in oncology (10, 11). This framework has also been applied to the use of telehealth in the unhoused population to investigate the effectiveness of various delivery models of primary care to people experiencing homelessness in England (12). The RE-AIM framework has demonstrated efficacy in these evaluation contexts.

## 2 Context

The CARES for the Unhoused program (CFU) serves the unhoused and those with unstable housing in Charleston, South Carolina, a small Southeastern city with limited infrastructure supporting the unhoused community including lack of Medicaid expansion leading to a significant number of uninsured individuals. CFU provides over 500 primary care visits for more than 250 unique patients each year with a demographic makeup (Table 1) that is mostly male (64.1%) and disproportionately African American (39%) compared with the South Carolina population (26%) (13) and predominantly uninsured (70.9%). CFU treats acute and chronic conditions and offers preventive care and specialist referrals as needed. The most common diagnoses treated are musculoskeletal conditions (22%), mental health conditions (21.7%) and cardiovascular conditions (21.3%). The program operates in partnership with two community-based organizations (CBOs) serving the unhoused and their respective staff social workers. Two attending family medicine physicians at the Medical University of South Carolina (MUSC) lead our program and oversee healthcare profession learners that include approximately 20 family medicine and internal medicine resident physicians per year and more than 100 MUSC medical and pharmacy students.

**Table 1:** Demographic characteristics of patient population.

Variable	Frequency	Percentage
Sex		
Male	143	64.1%
Female	80	35.9%
Race		
Black or African American	87	39.0%
White or Caucasian	109	48.9%
Other	5	2.2%
Unknown	22	9.9%
Ethnicity		
Hispanic or Latino	2	0.9%
Not Hispanic or Latino	190	85.2%
Unknown	31	13.9%
Age		
0–17	4	1.8.%
18–39	50	22.4%
40–59	114	51.1%
60 and up	55	24.7%
Insurance		
Private	11	4.9%
Medicaid/Medicare	54	24.2%
Uninsured/Self-Pay	158	70.9%

## 3 Key programmatic elements

Our program consists of three arms: (1) in-person visits at a CBO, (2) video visits at a CBO, and (3) street medicine outreach with CBO staff. For our clinic in-person and video visits we partner with The Navigation Center (TNC) which is a full spectrum drop-in social resource center focused on those experiencing homelessness. TNC provides physical clinical space to conduct visits. For telehealth visits, pre-professional students are in-person at TNC and facilitate visits between patients and remote attending physicians. For street medicine outreach, MUSC physicians and learners partner with TNC, the local homeless shelter (One80 Place), the Department of Mental Health and Emergency Medical Services. In person clinic and street medicine visits are conducted by resident physicians with either in-person oversight or tele-precepting. Tele-precepting is when a remote attending physician supervises healthcare profession learners that are in the same location as the patient, incorporating audio and video interface via cell phone, tablet or computer. The Doxy.me HIPPA compliant platform is used for video visits and tele-precepting on a laptop or tablet for both clinic and street medicine. Over 150 learners participate in the program annually. The program hours are 9 AM-5 PM every Tuesday and Thursday, a schedule developed with input from all stakeholders. Each day of clinical operations consists of a combination of each of the three visit types, with proportions based on the needs identified that day.

Our team completed a qualitative implementation evaluation of the CFU program using the RE-AIM framework measuring Reach, Effectiveness, Adoption, Implementation and Maintenance (9). This study was recognized as Quality Improvement by the MUSC IRB. Key stakeholder interviews were completed from January 2024 to May 2024 with patients (*n* = 9), CBO staff (*n* = 3) and learners (*n* = 7) to gain multidisciplinary perspectives on program moderators. Key stakeholders that were invited to participate were identified for CBO staff based on current staff members working in collaborating CBOs that had interacted with the CFU program. Healthcare profession learners were identified by having volunteered with the program in the past year. Patients were identified by having been seen for a medical visit by the program in the past year. Patient interviews were conducted by medical students that were not part of the healthcare team. Other key stakeholder interviews were conducted by a research coordinator not involved in program implementation. Interviews were completed in patient care sites including at the community organization facilities and street medicine outreach sites as well as virtually and lasted from 5 min to 30 min. All interviews were transcribed verbatim. Two trained coders utilized a template analysis approach (14, 15) in Microsoft Word, with an initial codebook derived from the RE-AIM framework to code the first set of two interviews. Template analysis utilizes a structured (“template”), yet flexible coding process for analysis of qualitative data. Since the interview guides in this study were based on the RE-AIM framework, template analysis allowed researchers to utilize an *a priori* template, based on the domains of the RE-AIM framework, while also allowing codes to be updated in the coding process. The codebook was updated in a dynamic fashion, allowing additional codes to emerge from the data. After each round of coding, code definitions were refined and coding discrepancies between the coders were discussed to arrive at consensus. After coding was completed, themes were summarized and compared by role (patients, CBO staff, learners).

## 4 Discussion

An implementation evaluation of the CFU program was conducted using key stakeholder interviews including healthcare profession learners, CBO staff, and patients seen in different care settings to identify barriers and facilitators to reach, effectiveness, adoption, implementation and maintenance, based on the RE-AIM framework (9). Interview themes were similar by role and identified barriers and facilitators to implementation outcomes of the program, based on RE-AIM. See Table 2 for illustrative quotations. Identified program moderators can be utilized to enhance future adaptations of medical programs for underserved, vulnerable populations.

**Table 2:** Exemplary quotations by RE-AIM domain.

Themes	Patient	Learner	Community stakeholder
Reach			
Opportunity to improve reach	“Awareness is a big one…” “The people will come to where you are…instead of moving all over…Get one, get a couple of areas where they know you are going to be at certain times. Then they’ll come to you. The ones who want the help, you know…”	“…selling it as something that does help…just to know the community better and know what we can offer patients would also get people more interested, apart from just the pure volunteerism of it. It also has practical implications for our practice.” (resident)	“I think just trying to encourage [patients] for their general wellness…” “…many times, as long as it’s somebody I’ve built rapport with and I trust, they’ll allow me to bring a doctor out. We operate under the assumption that it is them allowing us to provide care to them.”
Facilitators/barriers to reach	“They have to want it. Drugs is the issue for most homeless people, alcohol; they do not want to give it up. Cannot be helped. You have to help yourself.” “Coming here, knowing I do not want to go back to the life I was living, these people really helped change the path I’m on….”	“I think a lot of people hear street medicine and fear, what are they putting themselves in the middle of, Is it going to be dangerous? Am I going to be interacting with potentially dangerous people?” Because I think so many people in the medical student position just have not had exposure to this community.” (student)	“Sometimes we get a tip from other people in the community that something more severe might be going on…then there’s more trust to be built…A lot of times, it’s communicable diseases. There’s a lot of shame and stigma especially with HIV. Despite a lot of resources and a lot of great people working in that field right now, it can be really tough to accept that help, especially when you are so vulnerable.”
Effectiveness			
Perceived benefits	“I enjoyed it. They talked to me and, you know, advise, get me to understand what’s going on here. They help me a whole lot.” “…the just reliability of it. I know every time I come here they are going to see me and they are going to help me.” “It has gone from helping me get off of an addiction to physical ailments, to my mental wellbeing.” “…if you reach out for help, you have got to try your hardest to listen to them and do what they [say]…they are here to help you, not hurt you.”	“It’s definitely a rewarding experience… personally fulfilling and made me feel like my job was making a difference and that I was having some purpose.” (resident) “…I’m seeing a direct impact of what I’m doing and how I’m caring for people who otherwise would not have had a way to get that care.” (resident) “The program has completely shaped me into a wonderful, well-rounded physician… every single one of my attendings are super positive and love to teach.” (resident) “…it has allowed me to remember why I wanted to go into healthcare.” (student)	“They know they have a place to come to and get some of the immediate needs they need, or at least to be able to talk to somebody, to check in. The clinic, obviously, is amazing, and then our peer support that we have with mental health and then the mental health services we have…It’s quite welcoming, I think, to meet them where they are.” “It’s providing really necessary healthcare for people who have limited access to it. So, it’s a really huge impact.”
Unintended consequences	“…no negative. Every time I come here the staff and the Center’s very helpful.” “No, it has not had any negative. It’s all been positive to me. If you want it, you have got to work with it, too.” “…at first a little negativity…But after a little while all that just fell out the way. Being street people, you are going to hear different things about [different people]. And that can put some negativity on someone who is really trying to help you.”	“The only negative thing that I witnessed or I’ve heard about is some people feel unsafe going to the homeless camps, which I think that has been addressed.” (resident) “Maybe being exposed to things that are negative, but that just happens any time you are in a patient encounter that if they have hard things going on.” (student)	“We cannot keep up with all of the requests. A lot of the calls that are coming in, we can only do so much, and that, to me, is a negative, that we cannot keep up with the pace of the folks…we are not able to meet all of the needs and all of the people that want help.” “Sometimes, it can get rough here, but most of the time you just have in your head that this is their unfortunate journey, and we just have to be there and support them, and we pray for them.”
Opportunity to improve efficacy of care	“Maybe coming out more often.” “…just more resources…” “…add a little more volunteers to [the program].	“There was a lack of testing and labs and medications sometimes…the tangible resources would probably be the one limitation.” (resident) “If we had more information up front…I did not have any idea what I was getting into…more up front education about what kind of issues we would be seeing might better set us up for success…” (resident) “Exposure…you just have to go in with an open mind and be exposed. I do not think there’s any other way to build confidence in this type of population without being around this type of population.” (resident) “At the very beginning I was nervous, not really knowing what to do and what to expect and where my role would be. After going a few times, I know what should be done, what’s expected.” (student)	“We need a clinical social worker, actually, so I would say funding would be the biggest need right now for us so that we can have those folks in here, helping us support those efforts to keep up with the pace.” “I have often found that if I have a patient need, [the program] will always get somebody there. So, the number of patients I have found to be no barrier at all. We’ve gone into very large camps [to see patients]. There’s always been time to see, upwards of 10 people living in an encampment.”
Adoption			
Patient-provider communication	“… the team was great and everything. Very informative, and, you know, you have got to listen completely all through it, you know? If you have got any questions, you have got to clarify it…they’ll give me an honest answer.”	“I think how important it is to build rapport with that patient population… it means so much more when you actually see them and you talk to them and when you remember their names, what kind of impact that has on them.” (student) “This community tends to be wary of outside people coming in because you do not know someone’s motives or intentions when they are coming into your space…Authority figures can be threatening …I’ve been really impressed and surprised by the relationships that the staff involved in the clinic have been able to foster, to where [communication] has not ever been an issue.” (student)	“It’s a very unique circumstance to be able to interface with people who are in the situation that they are and not see them as that situation, but to see them as a human and treat them with dignity and respect.” “…it’s about building that rapport with them. The families love [this program]. I mean, I would love to expand that.”
Team communication	N/A	“The closed-loop communication is good, even if borderline redundant, I feel like it makes it more effective.” (resident) “I think students have to be comfortable with trying things and knowing that you are not immediately going to be good at them, but that’s why you have residents and others to assist. As long as students are encouraged and willing to ask questions, that’s the biggest thing, is just being willing to do and being willing to ask.” (student)	“The key to the success of what we do is that we are case-managing alongside the doctors and even mental health…We want to know what’s going on.” “You need to be prepared to have each other’s [team members] back…building that team trust is huge. So…working with folks that you come to know and come to trust in situations that can change very rapidly, obviously. It lets everybody do better work.”
Trust	“I always talk to [the program lead doctor]. If there’s an issue, I’ll bring it up, but I think she knows everything going on with me, so I do not have concerns. I do not know how she got involved on this level, but she did, and I’m grateful for that. And I will not do anything other than [what she says].” “I trust them…that’s why I come down here. But it’s up to me. And I know they are here on certain days. It’s up to me to get here.”	“…the openness of everyone there and just how much everyone trusted the program, I think would be the biggest things that surprised me…” (resident) “I think the teaching skills of the medical team have been phenomenal…they have been very helpful and very willing to ask questions and teach as needed without being overbearing.” (student)	“I love the way the residents are eager to learn. Some of them are deer-in-headlights. I can see it’s kind of scary sometimes, but it’s great to see compassion of these young doctors-to-be that want to help and be there, and eager to help a lot of folks that we have here.” “The way that rapport is built and maintained through street medicine, trust is huge. We’re going into where people live, often in very vulnerable situations.”
Coordination of care	“…you do not have a car [to seek medical help]…I can understand how the various services come together and work together and you have a social worker and [other staff]. So I’m appreciative of it, and I do not want to abuse the system in any kind of way…getting my seizure medication is important.”	“We are all very cohesive and work well together…” (resident) “…the street psychiatry aspect is important…Once I wanted to go out in the afternoon [to see patients] and then we had people from the local mental health centers coming out with us…that was an excellent opportunity.” (resident)	“Even on odd hours, I’ve been able to get hold of someone for a quick text consult or telehealth link so we can make sure we are getting access to care. It’s nice to have a deep bench of available people. Medical students coming into the field doing eye tests is incredible to add onto doctors and residents doing care. It’s nice to be able to go out and offer a full range of services.”
Implementation			
Access	“Anytime I’m not feeling good, I come and the door’s open and they give me a time to come back to recheck me and everything.” “And it probably cuts out a lot of emergency room visits for you guys. When people get hopeless, they have nowhere to go, then rely on the hospital.” “Maybe come out there more often. And people will know you. They can depend on you to be at a certain place at a certain time. That’s the only thing…”	“That’s what shocked me, we have a good amount of resources that we offer, it’s well set up, and we get people to close the loop so things do not get missed.” (resident) “…being part of this program I saw that there are more people than I thought that really do want healthcare, but there’s no means of getting it to them…some of my frustration was that sometimes I still struggle for pathways to get people help when they are having these resource limitations.” (resident)	“I do not think there’s anybody who has been actively wanting to see a provider that has been told they have to wait an amount of time…everybody that I’ve interacted with has gotten care fast enough that they have been satisfied.”
Follow up	“I got medicine I got to pick up now. They got the medicines that I need, but it’s up to the individual to go get the medicine…I got to come back here to get it tomorrow…so they help you get the medicine.”	“…it’s hard for people to get transportation to get labs. The majority of them even walk, or take the bus, and cost is a barrier, but we do our best to try to aid…the team [tries to] coordinate something.” (resident) “I had to run to the pharmacy and pick up some medications for people or another provider would pick up meds and we could distribute when we went out.” (resident)	“…being able to provide labs and imaging at low or no cost is great. It’s wonderful.” “…especially clients that do not have a phone, it can be nerve-wracking for some …they are afraid of going into a medical building for bloodwork….[the doctors are] great about getting costs down when needed, when things are too expensive we are able to cover some costs.”
Maintenance			
Factors promoting sustainability	“Just because it’s consistent and reliable and dependable…”	“It is well-integrated. It’s part of our curriculum…if it was more voluntary, I think you would not have that much exposure because our hours are so difficult.” (resident)	“…the team’s flexibility to go where we need them, when it’s best for the client.it puts the client back in the driver’s seat, which is something they do not get to experience a lot with how they are living.”
Outreach efforts	“…social work, from the aspect of helping people with housing and food.”	“…I would [work] with a clinical psychologist or psychiatrist, a behavioral health provider. That was super helpful…I think a wound care provider could be very helpful.” (resident)	“…[patients] live outside, they are doing something to survive, trying to build a shelter or something happens…twist a shoulder the wrong way, tweak a back, and then they get laid up in the tent for a couple of weeks until we come across them and try to get them some help… physical therapy would be helpful.”

### 4.1 Reach

#### 4.1.1 Opportunity to improve reach

Optimizing reach, and in this case the number of individuals served by a program, is central to maximizing positive impact. Patients within the CFU program were seen both at TNC and in the unsheltered community locations where they reside. Patients reported initiating participation in the program following hospital admission, being brought to TNC by someone, and having an outreach provider approach them. Patients suggested opportunities to improve reach by recruiting new patients including creating and promoting more awareness of the program via word of mouth, providing handouts, being present at events that provide meals, and visiting additional areas where the target population lives (parks, etc). CBO staff described the importance of encouragement and trust in reaching more patients and treating patients with high levels of respect to establish rapport. The importance of collaborations with other organizations that support this population was also a strategy suggested to reach more patients.

Strategies to enhance reach to recruit more learners (students, residents) included education on the positive learning effects of the program and future clinical career benefits. This increased awareness could be accomplished during orientation activities, and by ongoing informational flyers, email communication from medical university program directors, word of mouth from other residents and students that participate in the program, and communication from an appointed liaison between CFU and the medical university.

#### 4.1.2 Facilitators/barriers to reach

Patients conveyed the need to have an innate desire to receive medical care, as someone that is averse to receiving medical care or adhering to medical recommendations will be difficult to reach. People who use substances and those with communicable diseases were mentioned as subpopulations that may be particularly difficult to reach due to fear of stigma associated with these conditions and concurrent patient reluctancy to receive care. However, it was suggested that building rapport with these patients has proven successful in facilitating trust and ultimately delivering any needed health care. Patients reported a common barrier to expanding treatment reach included lack of transportation to complete diagnostic workups such as labs or imaging, which is challenging since often these specific services cannot be delivered to them in the setting in which they reside.

In discussing willingness to participate in the medical program, students reported a common barrier to participation is their general lack of education and experience with the unhoused community and population, leading to fear of providing care in a potentially dangerous situation. Residents and students also reported that participating in this program was at times challenging with their existing schedules and that incentives to volunteer could be favorable to eliciting more participation.

### 4.2 Effectiveness

#### 4.2.1 Perceived benefits

Describing positive and negative outcome effects of an intervention, including potential impact on quality of life, is vital to the understanding of comprehensive intervention effectiveness. By defining individual differences in intervention effectiveness, strategies can be created as opportunities to improve intervention efficacy of care.

All patients expressed appreciation for the program overall and specifically for provider reliability and the ease with which they could receive care in the setting where they reside or were already receiving services, with no additional need for travel. Patients noted that the providers really listened to them and were concerned, caring, and understanding and identified these as essential components of care delivery. One patient explained that he knew the provider was trying to help, so he was motivated to do his best to do everything the provider suggested. Benefits of the program that were discussed included support to end a substance addiction, gaining access to needed medications, and improvements in mental and physical health. Patients described the program as “helpful and wonderful” and one patient described the improved quality of life experienced after this program as he stated his “perception of life has changed….”

CBO staff described some of the multi-faceted benefits of the program, most notably offering essential comprehensive health care for unhoused individuals, who have limited access to care. The care can be obtained quickly and is flexible since it is provided in the setting where individuals reside or at a CBO where they are already seeking social services. The program was described as providing a source of comfort to this population, which they do not often experience from their lifestyle. CBO staff also discussed the benefits of the program to learners, as they can gain experience with this medical setting and population.

Similarly, learners stated they were able to learn more about resources for the underserved and unhoused communities through participation in this program, allowing them to feel more “well rounded” in their career training. They described their role in the program as “rewarding and fulfilling” as it provided tangible reminders and motivation of their desire to help others and enforced the reasons they chose a career in the health care field. Learners enjoyed seeing the direct impact their participation had, as they were able to provide health care to those that may not otherwise have had access to medical services. They also appreciated the welcoming and enthusiastic response they received from unhoused patients with whom they engaged. For learners, providing care to this population demonstrated gaps in health care services and encouraged one resident to commit to continue to consider these gaps and implications for health care policy in the future.

#### 4.2.2 Unintended consequences

It is important to identify negative or unintended consequences of interventions to help devise strategies to overcome these negative effects and maximize intervention adoption and sustainability.

One patient disclosed that they felt some personal negativity related to the program initially, likely due to negative discussions with others. However, this patient reported their negativity quickly dissolved and was unwarranted as they learned the providers intent was to deliver beneficial medical care. All of the other patients reported no unintended consequences or negative effects from the program, while some additionally emphasized the positive and helpful aspects of the program.

One resident and one student both described safety concerns related to the setting of this program, although both agreed the medical university had recently addressed this by starting initial triage for setting safety with outreach workers familiar with specific unhoused community camps. The medical university program sponsors also emphasize the optional nature of visiting specific sites based on individual comfort level of learners. Similarly, one CBO staff member described the occasional “rough” setting in which care is provided on the street to this population. Yet, all stakeholders emphasized gratitude for the support the program provides to the unhoused population.

#### 4.2.3 Opportunity to improve efficacy of care

Patients only discussed a few opportunities for improving the efficacy of program care, which included more frequent visits with providers and additional program workers to expand program scope.

CBO staff agreed that additional program workers would be beneficial, including a program specific case manager or social worker. While one CBO staff member stated that they have always been able to have the program provide visit patients when requested, it was agreed that to have capacity to treat the increasing patient case load and expanding community locations additional staff will be increasingly needed. Additional clinical private space for exchange of health information was also identified as a potential area of need.

One healthcare professional learner discussed the program could benefit from additional resources (availability of on-site lab testing, affordable medications) to meet the increasing patient case load and complex medical needs. Other learners described the need for additional initial education prior to working with the unhoused population to have a more clear understanding of the unique challenges this population experiences, including basic education on housing infrastructure, medication costs, insurance, and addiction treatment. Efficiency challenges with EMR documentation related to lack of administrative support for the walk-in nature of appointments was also cited by learners. However, they also described the ability to learn processes quickly and gain confidence in their skills in this setting and appreciated the opportunity for autonomy, while having oversight and mentorship by the lead program attending physicians. Learners generally felt that after working a few shifts for this program, they felt more comfortable in the setting. Learners cited increasing interdisciplinary team members involved such as nursing and pharmacy students and mental health providers, would benefit the program and patients.

### 4.3 Adoption

#### 4.3.1 Patient-provider communication

Communication is central to the patient-provider relationship in the healthcare setting. Patients overwhelmingly reported that the medical team communicated with them in a way they were able to understand. The importance of listening, not rushing, and limiting use of technical terms during medical communication were identified as facilitators to improved communication. One patient specifically identified the importance of the way questions were asked as a factor which could either promote or impair effective communication.

Learners and CBO staff identified the importance of building rapport with patients when providing care and the importance of developing relationships over time, with a focus on individualized interactions based on respect. A student identified the concern for distrust of authority figures in this population and the need to foster relationships to limit the impact of this potential barrier.

#### 4.3.2 Team communication

Interdisciplinary team communication was discussed with learners and CBO staff and included communication practices among health professions students, resident physicians, attending physicians, and CBO staff. Team communication was generally reported as effective and need for open communication was accentuated along with need for flexibility, trust, support and acceptability amongst all team members. The program learning environment was reported as open and inviting, and new learners reported initial benefit from receiving additional information and communication with more experienced team members. Learners reported attending physician supervision was available when needed with both in person and telehealth based precepting modalities. There was a specific theme discussed among learners related to “closed-loop communication,” which included informing members of the care team who were not present for the patient encounter of their responsibilities for patient care follow up. This was reported as effective, yet occasionally redundant leading to increased work load. Strategies to streamline follow up communication to limit burden for team members are important to sustain efficacy.

#### 4.3.3 Trust

Trust was an important theme across multiple axes in the interactions between key stakeholders. Patients strongly reported trust in the medical recommendations of the care team, however; they also identified the individual motivation needed to seek help as part of the process of accessing care. While patients directly mentioned trusting the recommendations of the program medical director, one patient stated they were still attempting to find trust in the hospital system. Trust among medical team members (students, residents, and attending physicians) was highly reported and the importance of an interdisciplinary team and leveraging of community resources was identified as critical to providing care. Finally, the importance of building and maintaining trust with patients was identified by two CBO staff members as essential to supporting unhoused individuals who may fear being stigmatized and who are often in vulnerable situations.

#### 4.3.4 Coordination of care

Coordination of care was discussed by patients, learners and CBO staff. There was a focus on coordination of program medical care with mental health services and social workers and the need for flexibility with time and location to see patients since patients are potentially treated outside of typical business hours and in their own environment. CBOs directly and indirectly working with the CFU program as well as hospital systems were mentioned as integral to facilitate this coordinated care as was the closed loop communication described above.

### 4.4 Implementation

#### 4.4.1 Access

Factors relevant for access to program care included frequency of encounters and clinic sessions as well as consideration for whether program availability was adequate to meet patient needs. Patients appreciated the consistency of the timing of clinic sessions with regard to available days and times. However, some patients did report potential benefit of increased options for times in clinic or additional time slots and continued expansion of access to resources to support patient needs. Learners noted the clinic has expanded over the course of the year and some learners were surprised by the size of the population served by this program, although also noted needed for additional expansion to meet growing patient demand. CBO staff believed that patients were typically seen within adequate amounts of wait times and reported the clinic walk-in structure without direct appointments had not resulted in prohibitive wait times.

#### 4.4.2 Follow up

Patients reported that they were able to obtain follow up care including medication, lab work, and other services with few limitations. At times, accessing these follow up services required a return visit to TNC. Patient reported barriers for follow up services were transportation and more expensive medications without more affordable alternatives. Learners commented specifically on the limitations of transportation for lab work and medications and also noted that screening recommendations were less likely to be recommended or completed within this program. CBO staff appreciated the opportunity for patients to receive labs and imaging but identified that increased access to a variety of services and available care locations would be beneficial due to transportation barriers. Patients who may not have regular phone access were more likely to have challenges with follow up services. CBO staff commented on efforts of both TNC and MUSC to reduce costs and assist in transportation when possible.

### 4.5 Maintenance

#### 4.5.1 Factors promoting sustainability

Patients endorsed that they would continue to receive care from the clinic, based on perceptions of consistency, reliability, dependability, and availability of the care provided. From a learner perspective, integration in the curriculum was noted as a facilitator. Others discussed intention for continued participation due to flexible program specific work hours. CBO staff discussed the sustained benefit of the clinic as a flexible option for patient care, outside of emergency care, that offers team-based outreach with flexible locations.

#### 4.5.2 Outreach efforts

All stakeholders identified the potential benefit of additional outreach services including ophthalmology, dental, physical therapy, and mental health including psychiatry. In addition, opportunity for additional onsite or outreach services, including labs, was identified. Additional student services including physical therapy were identified as potential new multidisciplinary team members.

## 5 Limitations

There are a few constraints for this implementation study. There is the potential for response bias as individuals more likely to continue to receive care may be more likely to volunteer to participate in interviews about the program. Although interviews were not conducted by or in the vicinity of residents or attending physicians and were conducted by student volunteers not actively engaged in the care team at the time of the interview, the interviewers seen alongside the outreach team at times may have impacted perceived roles during interviews and the expressed opinions of patients. Due to the nature of the unhoused population, those that are more difficult to reach were inherently not included in interviews. Finally, the data collection focused on obtaining interviews from a variety of stakeholders limiting the number of individuals interviews within each group which can impact the sample size and representativeness of each group. We believe the consistency of themes across groups supports the themes and conclusions from qualitative interviews.

## 6 Conclusion

This implementation evaluation of the CFU program described the benefits and moderators of this innovative care model. The program provides convenient comprehensive medical care to the unhoused population using a hybrid model of telehealth and in-person care while concurrently providing needed hands-on educational opportunities for health professional learners. For long term improvement and sustainability of the program, it is necessary to have adequate resources, staffing, and space to meet increasing patient demand as well as added care involving additional areas of specialty to improve the breadth of services. For learners, it will be important to continue to adapt their educational schedules to allow for program participation while also providing initial training and education on the unique aspects of treating this population. Based on the results of this study, recent efforts have centered around increasing point of care lab testing, enhancing access to cancer screening and other preventive health measures for patients, enhancing preparatory materials for learners, and further partnering with community-based organizations to expand staffing and the variety of services offered. To continue to provide the highest level of quality care, it is vital to maintain open communication among all program team members and patients as well as coordination of care with health care and community- based systems. The identified facilitators and barriers can be utilized in the future to modify and adapt program components to expand equitable health care access to a vulnerable population.

## Data Availability

The original contributions presented in the study are included in the article/supplementary material, further inquiries can be directed to the corresponding author.
